# Vaccination Intentions During Pregnancy and Among Parents of Young Children

**DOI:** 10.1001/jamanetworkopen.2025.20667

**Published:** 2025-07-01

**Authors:** Lavanya Vasudevan, Rachael M. Porter, Walter A. Orenstein, Tara M. Vogt, Robert A. Bednarczyk

**Affiliations:** **Author Affiliations:** Hubert Department of Global Health, Rollins School of Public Health, Emory University, Atlanta, Georgia (Vasudevan, Porter, Orenstein, Bednarczyk); Department of Medicine, School of Medicine, Emory University, Atlanta, Georgia (Orenstein); National Center for Immunization and Respiratory Diseases, Centers for Disease Control and Prevention, Atlanta, Georgia (Vogt).

## Introduction

Many parents in the US choose to delay or refuse vaccines that are recommended for their child from birth to age 18 months.^1-4^ Research is necessary to understand the value of intervening during pregnancy to proactively support parents with vaccination decisions before the birth of the child, as implementation of such interventions will require substantial engagement of health care professionals and entities outside of the pediatric care setting. Therefore, 2 concurrent national cross-sectional surveys were conducted in April 2024 to describe vaccination intentions during pregnancy and vaccination behaviors after the child’s birth for all vaccines recommended for children from birth to age 18 months.^1^

## Methods

This survey study followed Strengthening the Reporting of Observational Studies in Epidemiology (STROBE) reporting guidelines. Participants were recruited from a nationally representative panel of US adults with a sampling frame covering nearly 100% of US households.^5^ Eligibility was limited to English language survey takers and those aged 18 years and older who self-reported pregnancy and parent status. Eligible panel members received the survey link from the panel provider. Those opting in after reading the implied consent script self-administered the survey using an online survey platform. Authors retained full ownership of the data. Participants who were pregnant were asked about vaccination intention for their child after birth with the question: “Will your child receive any vaccinations after birth?” (with response options of “yes,” “no,” and “I don’t know”). Those responding yes were asked, “Do you plan to refuse or choose not to get 1 or more recommended vaccines (including seasonal flu or COVID-19 vaccines) for your child after birth?” followed by, “Do you plan to delay or space out ANY recommended vaccines (including seasonal flu or COVID-19 vaccines) for your child after birth?” (responses for both questions were “yes,” “no,” or “I don’t know”). Parents were asked the same questions rephrased to assess actual vaccination for their youngest child. Descriptive analyses were conducted in SAS version 9.4 (SAS Institute), with applied geodemographically calibrated sample weights. A 6-level outcome variable was created to describe vaccination decisions: (1) accept all vaccines, (2) delay some or all vaccines, (3) delay some and refuse some vaccines, (4) refuse some vaccines, (5) refuse all vaccines, and (6) undecided (eTables 1 and 2 in Supplement 1). The study protocol was approved by the Emory University institutional review board.

## Results

A total of 174 pregnant participants (182 surveyed [95.6% completion rate]; 128 aged 30 years or older [64.0%]; 34 Hispanic [27.0%], 17 non-Hispanic Black [14.4%], 112 non-Hispanic White [52.9%]) and 1765 parents (1874 surveyed [94.2% completion rate]; 1488 aged 30 years or older [78.1%]; 300 Hispanic [21.7%], 154 non-Hispanic Black [11.4%], 1174 non-Hispanic White [56.0%]) completed the survey. Among pregnant participants, 68 pregnancies (37.6%) were nulliparous (Table). Approximately half of participants had a bachelor’s degree or higher (103 pregnant participants [49.4%]; 1041 parents [45.1%]); more participants were currently married vs not (117 pregnant participants [69.0%]; 1316 parents [75.6%]) and resided in an urban vs rural or suburban areas (138 pregnant participants [77.6%]; 1380 parents [79.5%]).

The proportions intending to accept or accepting all recommended vaccines for children were similar among pregnant participants and parents (Figure). The proportion intending to refuse or refusing some or all vaccines for their child (without delays) was lowest among nulliparous pregnant participants (5 of 68 [4%]) and highest among parents (391 of 1765 [33%]). Uncertainty about childhood vaccination was highest among nulliparous pregnant participants (31 of 68 [48%]) and lowest among parents of young children (78 of 1765 [4%]).

## Discussion

Given the high decisional uncertainty during pregnancy about vaccinating children after birth, there may be value in intervening during pregnancy to proactively support families with childhood vaccination decisions. Strengths of our study include the national scope of the surveys. Study limitations include the inability to confirm concordance between vaccine intentions during pregnancy and future childhood vaccination behaviors, the potential influence of prior vaccination decisions for older children, the small counts in some response options that may create unstable estimates, and differences in demographic characteristics between samples. Future interventions should account for differences in uptake of seasonal (ie, COVID-19, influenza) vs routinely recommended vaccines. Future studies with longitudinal follow-up may shed further light on evolution of vaccination decisions from pregnancy to parenthood, and the effectiveness of intervening proactively during pregnancy.

## Supplementary Material

Supplement 2 Data Sharing Statement

Supplement 1 **eTable 1.** Vaccine Decision Outcome Variable Categorization, by Vaccine Intention Questions Asked in a Survey of Participants Who Are Pregnant
**eTable 2.** Vaccine Decision Outcome Variable Categorization, by Vaccine Behavior Questions Asked in Survey of Parents of Young Children (0-5 Years)

## Figures and Tables

**Figure.**
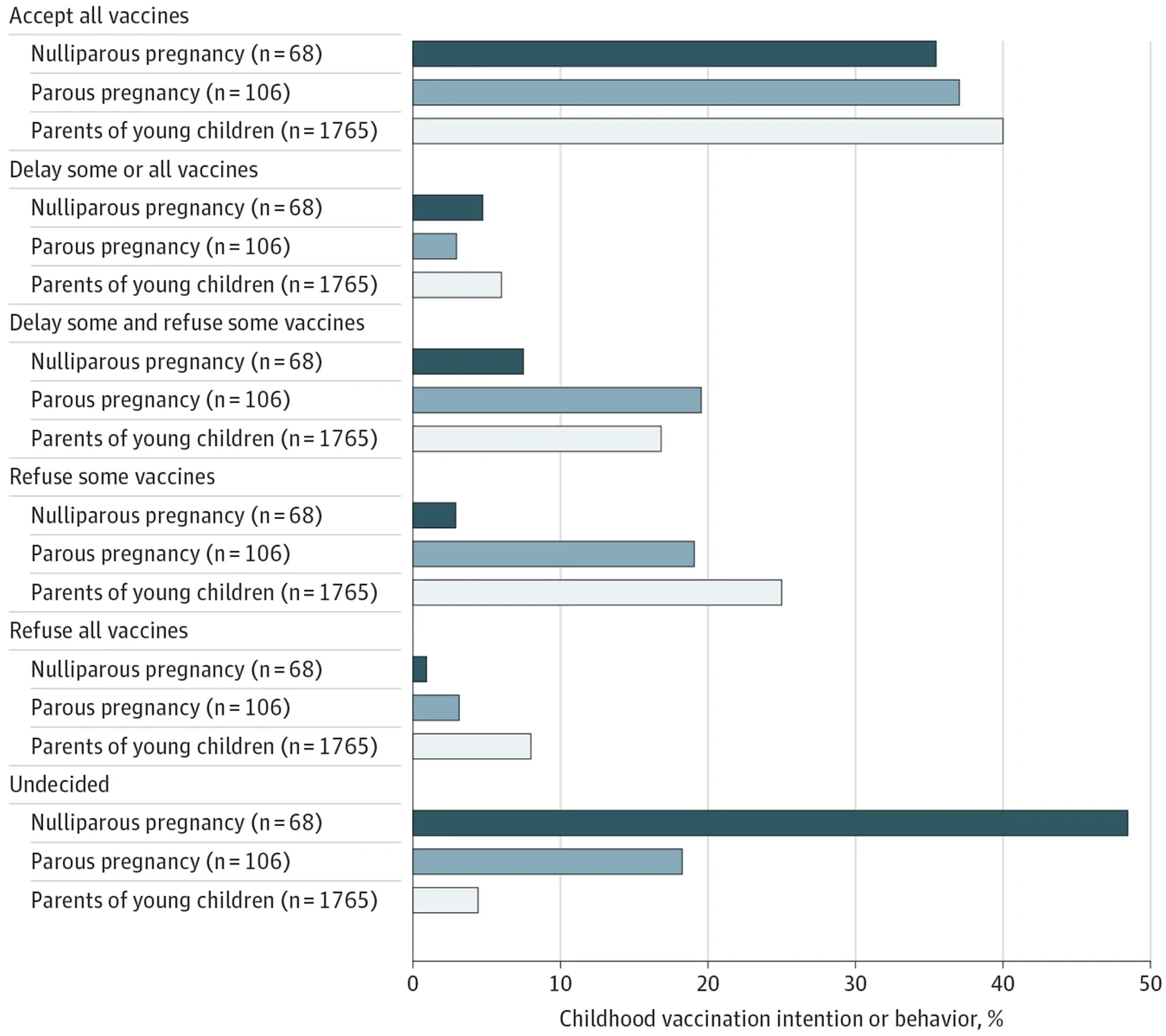
Childhood Vaccination Intention by Parity and Childhood Vaccination Behaviors Reported by Parents

**Table.** Characteristics From 2 Concurrent National Surveys Conducted Among Participants Who Were Pregnant and Parents of Young Children Aged 0 to 5 Years

Characteristics	Participants, unweighted No. (weighted %)^a,b^	
	Pregnant (n = 174)	Parent of young children (n = 1765)
Trimester of pregnancy^c^		
First	60 (30.7)	NA
Second	60 (37.5)	NA
Third	53 (31.4)	NA
Pregnancy parity		
Nulliparous	68 (37.6)	NA
Parous	106 (62.4)	NA
Age of youngest child, y^d^		
0-2	NA	904 (54.2)
3-5	NA	847 (45.3)
Age, y		
18-29	46 (36.0)	277 (21.9)
≥30^e^	128 (64.0)	1488 (78.1)
Education level		
Less than high school	8 (9.2)	71 (6.4)
High school	26 (20.1)	220 (24.2)
Some college	37 (21.3)	433 (24.2)
Bachelor’s degree or higher	103 (49.4)	1041 (45.1)
Race and ethnicity^f^		
Black or African American, non-Hispanic	17 (14.4)	154 (11.4)
Hispanic	34 (27.0)	300 (21.7)
White, non-Hispanic	112 (52.9)	1174 (56.0)
≥2 races and other, non-Hispanic	11 (5.7)	137 (10.9)
Household income, $		
<75 000	79 (39.0)	746 (36.1)
≥75 000	95 (61.0)	1019 (63.9)
Marital status^g^		
Currently married	117 (69.0)	1316 (75.6)
Not currently married	52 (27.6)	449 (24.4)
Current employment status		
Working full-time	94 (54.0)	1040 (66.5)
Working part-time	29 (16.1)	289 (12.2)
Not working	51 (29.9)	436 (21.4)
Residence		
Urban	138 (77.6)	1380 (79.5)
Rural and suburban	30 (22.4)	331 (20.5)

Abbreviation: NA, not applicable.

a Unweighted values represent actual numbers reported in dataset.

b Weighted percentages calculated using geodemographic weights obtained from the panel provider.^5^

c Trimester of pregnancy unknown for one participant reporting pregnancy.

d Age of youngest child unknown for 14 participants who said they were parents.

e Age categories for participants aged 30-44 years and 45 years and older were combined due to small cell size.

f Categories “Other, non-Hispanic” and “≥2 races, non-Hispanic” combined due to small cell size. Races included in “Other, non-Hispanic” include: American Indian or Alaska Native, Asian, Native Hawaiian or other Pacific Islander, and different race. Race and ethnicity data were provided by the panel provider and not directly collected as part of the survey.

g Category for not currently married included widowed, divorced, separated, and never married.

## Data Availability

See Supplement 2.
