# Supplementary material for: Vaccination Intentions During Pregnancy and Among Parents of Young Children
Source: JAMA Netw Open. Author manuscript; Available in PMC 2026 Sep 17. (PMC13583879; doi:10.1001/jamanetworkopen.2025.20667)
Supplement: Supplementary file 1 [file NIHMS2206796-supplement-Supplement_1.pdf]

Supplemental Online Content

Vasudevan L, Porter RM, Orenstein WA, Vogt TM, Bednarczyk RA. Vaccination intentions during pregnancy and among parents of young children. *JAMA Netw Open.* 2025;8(7):e2520667. doi:10.1001/jamanetworkopen.2025.20667

eTable 1. Vaccine Decision Outcome Variable Categorization, by Vaccine Intention Questions Asked in a Survey of Participants Who Are Pregnant

eTable 2. Vaccine Decision Outcome Variable Categorization, by Vaccine Behavior Questions Asked in Survey of Parents of Young Children (0-5 Years)

This supplemental material has been provided by the authors to give readers additional information about their work.

© 2025 Vasudevan L et al. *JAMA Network Open*.

eTable 1. Vaccine Decision Outcome Variable categorization, by vaccine intention questions asked in a survey of participants who are pregnant.

	**Will your child receive any vaccinations after birth? (Yes, No, I don't know)**	**Do you plan to refuse or choose not to get one or more recommended vaccines (including seasonal flu or COVID-19 vaccines) for your child after birth? (Yes, No, I don't know)**	**Do you plan to delay or space out ANY recommended vaccines (including seasonal flu or COVID-19 vaccines) for your child after birth? (Yes, No, I don't know)**
**Accept all vaccines**	Yes	No	No
**Delay some or all vaccines**	Yes	No	Yes
**Delay some and refuse some vaccines**	Yes	Yes	Yes
**Refuse some vaccines**	Yes	Yes	No
**Refuse all vaccines**	No	n/a	n/a
**Undecided**[1]	I don't know	n/a	n/a
**Undecided**[1]	Yes	No	I don't know
**Undecided**[1]	Yes	Yes	I don't know
**Undecided**[1]	Yes	I don't know	No
**Undecided**[1]	Yes	I don't know	Yes
**Undecided**[1]	Yes	I don't know	I don't know

[1]Every instance of "I don't know" led to categorization as "Undecided." All possible combinations found in the dataset are listed here.

© 2025 Vasudevan L et al. *JAMA Network Open*.

eTable 2. Vaccine Decision Outcome Variable categorization, by vaccine behavior questions asked in survey of parents of young children (0-5 years).

	**Has your youngest child received any recommended vaccinations? (Yes, No, I don't know)**	**Have you ever refused or decided not to get one or more recommended vaccines (including seasonal flu or COVID-19 vaccines) for your youngest child? (Yes, No, I don't know)**	**Do you plan to delay or space out ANY recommended vaccines (including seasonal flu or COVID-19 vaccines) for your youngest child? (Yes, No, I don't know)**
**Accept all vaccines**	Yes	No	No
**Delay some or all vaccines**	Yes	No	Yes
**Delay some and refuse some vaccines**	Yes	Yes	Yes
**Refuse some vaccines**	Yes	Yes	No
**Refuse all vaccines**	No	n/a	n/a
**Undecided[1]**	I don't know	n/a	n/a
**Undecided[1]**	Yes	No	I don't know
**Undecided[1]**	Yes	Yes	I don't know
**Undecided[1]**	Yes	I don't know	No
**Undecided[1]**	Yes	I don't know	Yes
**Undecided[1]**	Yes	I don't know	I don't know

[1]Every instance of "I don't know" led to categorization as "Undecided." All possible combinations found in the dataset are listed here.

© 2025 Vasudevan L et al. *JAMA Network Open*.
