# Supplementary material for: Vaccination Intentions During Pregnancy and Among Parents of Young Children
Source: JAMA Netw Open. Author manuscript; Available in PMC 2026 Sep 17. (PMC13583879; doi:10.1001/jamanetworkopen.2025.20667)
Supplement: Supplementary file 2 [file NIHMS2206796-supplement-Supplement_2.pdf]

Data Sharing Statement

Data
Data available: Yes
Data types: Deidentified participant data
How to access data: Data is available upon request from Lavanya Vasudevan (lavanya.vasudevan@emory.edu)
When available: beginning date: 12-31-2028

Supporting Documents
Document types: None

Additional Information
Who can access the data: Data will be made available to researchers whose proposed use of the data has been approved.
Types of analyses: Any purpose
Mechanisms of data availability: Data transfer will occur after approval of the proposal and may require the establishment of appropriate transfer agreements and ethics review.
